# Lung cancer metabolomics: a pooled analysis in the Cancer Prevention Studies

**DOI:** 10.1186/s12916-024-03473-1

**Published:** 2024-06-24

**Authors:** Ziyin Tang, Donghai Liang, Emily L. Deubler, Jeremy A. Sarnat, Sabrina S. Chow, W. Ryan Diver, Ying Wang

**Affiliations:** 1 Gangarosa Department of Environmental Health, Rollins School of Public Health, Emory University, Atlanta, GA USA; 2 Department of Population Science, American Cancer Society, Atlanta, GA USA; 3 Barcelona Institute for Global Health (ISGlobal), Barcelona, Spain; 4 Universitat Pompeu Fabra (UPF), Barcelona, Spain

**Keywords:** Lung cancer, Etiology, Screening, Metabolomics, Sphingomyelin, Bile salt, Lipid metabolism, Amino acid metabolism

## Abstract

**Background:**

A better understanding of lung cancer etiology and the development of screening biomarkers have important implications for lung cancer prevention.

**Methods:**

We included 623 matched case–control pairs from the Cancer Prevention Study (CPS) cohorts. Pre-diagnosis blood samples were collected between 1998 and 2001 in the CPS-II Nutrition cohort and 2006 and 2013 in the CPS-3 cohort and were sent for metabolomics profiling simultaneously. Cancer-free controls at the time of case diagnosis were 1:1 matched to cases on date of birth, blood draw date, sex, and race/ethnicity. Odds ratios (ORs) and 95% confidence intervals (CIs) were estimated using conditional logistic regression, controlling for confounders. The Benjamini–Hochberg method was used to correct for multiple comparisons.

**Results:**

Sphingomyelin (d18:0/22:0) (OR: 1.32; 95% CI: 1.15, 1.53, FDR = 0.15) and taurodeoxycholic acid 3-sulfate (OR: 1.33; 95% CI: 1.14, 1.55, FDR = 0.15) were positively associated with lung cancer risk. Participants diagnosed within 3 years of blood draw had a 55% and 48% higher risk of lung cancer per standard deviation increase in natural log-transformed sphingomyelin (d18:0/22:0) and taurodeoxycholic acid 3-sulfate level, while 26% and 28% higher risk for those diagnosed beyond 3 years, compared to matched controls. Lipid and amino acid metabolism accounted for 47% to 80% of lung cancer-associated metabolites at *P* < 0.05 across all participants and subgroups. Notably, ever-smokers exhibited a higher proportion of lung cancer-associated metabolites (*P* < 0.05) in xenobiotic- and lipid-associated pathways, whereas never-smokers showed a more pronounced involvement of amino acid- and lipid-associated metabolic pathways.

**Conclusions:**

This is the largest prospective study examining untargeted metabolic profiles regarding lung cancer risk. Sphingomyelin (d18:0/22:0), a sphingolipid, and taurodeoxycholic acid 3-sulfate, a bile salt, may be risk factors and potential screening biomarkers for lung cancer. Lipid and amino acid metabolism may contribute significantly to lung cancer etiology which varied by smoking status.

**Supplementary Information:**

The online version contains supplementary material available at 10.1186/s12916-024-03473-1.

## Background

According to Global Cancer Statistics 2020, lung cancer accounts for 11.4% of the 19.3 million newly diagnosed cancer cases and remains the leading cause of cancer mortality [1]. Lung cancer is a heterogeneous tumor with several differentiation types. It is often diagnosed at an advanced stage and the 5-year survival rate is 24.6% [2–4]. The pathogenesis of lung cancer is believed to be influenced by gene-environment interaction [5, 6]. Variability in cellular, molecular, and genetic characteristics in lung cancer histological types has been well-documented [2]. Along with the change in the environmental and behavioral risk factors, the distribution of lung cancer displays great demographic, temporal, and geographical variability [7]. Surprisingly, epidemiological findings have shown that approximately 25% of lung cancer cases are not attributable to tobacco smoking, and the rate of lung cancer in never-smokers is increasing [8, 9]. Numerous studies have shown disparities in epidemiological, clinical, and molecular characteristics arising in smokers and never-smokers, indicating the possibility of distinct etiologies for the development of lung cancer in each group [8, 10]. A better understanding of the heterogeneity in lung cancer etiology has important implications in prevention, early detection and diagnosis, tumor classification, prognosis, and personalized therapeutic decision.

Over the past decades, metabolomics has emerged as a promising technique of studying the comprehensive metabolic profile in biospecimen, providing valuable information for the practice of precision medicine [11]. As substantially altered metabolism has been proven to be a hallmark in cancer cells [12, 13], the application of metabolomics in lung cancer provides an outstanding opportunity to elucidate the etiology and identify potential screening and early detection biomarkers. A growing number of metabolomics studies have examined lung cancer-driven metabolic changes in different biosamples [14]. Most studies have focused on characterizing the metabolic signatures differentiated by histological types in blood-based samples [15–17], while few have focused on stage-differentiated metabolic signatures [17–20]. Notably, these previous studies were mostly targeted metabolomics analyses, which focused on a limited number of metabolic endpoints. Overall, the existing findings display considerable heterogeneity among the studies. No metabolites were replicated and validated across studies, thereby limiting broad inference and the potential for their development as clinically applicable biomarkers [14]. To our knowledge, only one untargeted metabolomics application has been conducted in lung cancer research [21] and none have been performed in the USA.

To address these critical knowledge gaps, we conducted a comprehensive and exploratory metabolomics study on lung cancer within the Cancer Prevention Studies (CPS) [22, 23]. These well-constructed large prospective cohorts have pre-diagnosis samples with comprehensive information on lifestyle factors, and long-term follow-up provides a unique opportunity to better understand potential metabolic signatures in pre-diagnosis stage associated with lung cancer etiology.

## Methods

### Study design and population

Lung cancer cases and matched controls included in this analysis are participants from the CPS-II Nutrition cohort and ongoing CPS-3 cohort. At enrollment of the CPS-II Nutrition cohort in 1992–1993, participants completed a self-administered questionnaire that included anthropometric, demographic, dietary, lifestyle, and medical information. Follow-up questionnaires were sent to the cohort participants in 1997 and every other year thereafter to update exposures and to ascertain newly diagnosed cancers. A subset of 39,371 CPS-II Nutrition cohort participants provided a non-fasting blood samples between 1998 and 2001, and the information on demographic characteristics and other covariates in the analysis was assessed from the survey collected at blood draw or the 1999 survey. At enrollment of the CPS-3 cohort between 2006 and 2013, participants provided informed consent, a non-fasting blood sample and completed a brief enrollment survey on demographic characteristics and other covariates. Follow-up questionnaires were sent to active participants in 2015 and every 3 years to update exposures and ascertain newly diagnosed cancer cases. Detailed descriptions of the two cohorts can be found elsewhere [22, 23]. All aspects of the CPS-II Nutrition cohort (IRB00045780) and CPS-3 cohort (IRB00059007) were reviewed and approved by the Emory University Institutional Review Board.

A total of 1913 lung cancer cases were identified in the CPS-II Nutrition cohort through June 2015 and 176 lung cancer cases were identified in the CPS-3 Cohort through December 2015. Cases in the CPS-II Nutrition cohort were first identified through self-report and then were verified with medical records, state cancer registry linkage, or linkage with the National Death Index (defined by ICD-10 codes C33 and C34, excluding histology codes ≥ 9590). Cases in the CPS-3 cohort were identified primarily through linkage with state cancer registries, and a small proportion were identified by self-report that were verified by medical records during tumor collection. We applied a series of exclusion criteria to include participants (Additional file 1: Fig. S1). As a result, 500 and 123 lung cancer cases from the CPS-II Nutrition cohort and CPS-3 cohort were included in the analysis, respectively. Controls who were cancer-free at the time of case diagnosis were matched 1:1 to cases on age at blood draw (± 6 months), sex, race/ethnicity, and blood draw date (± 30 days).

### Metabolomics profiling

The pre-diagnosis blood samples collected from both cohorts were sent to Metabolon, Inc. (Durham, NC, USA) for untargeted metabolomics profiling simultaneously, using ultrahigh-performance liquid chromatography-tandem mass spectrometry (UPLC-MS/MS) analysis techniques. A detailed process was described elsewhere [24, 25] and in supplemental materials.

A total of 1,401 metabolites were detected. After filtering metabolites that were unknown (*n* = 238), were missing technical intraclass correlation coefficient (ICC) (*n* = 34), with ICC < 50% (*n* = 201), and were undetectable in > 90% of samples (*n* = 41), 887 known metabolites were included in the statistical analysis with an average ICC of 84% (interquartile range (IQR): 77–94%) and the coefficient of variation (CV)% of 24% (IQR: 12–30%).

### Statistical analysis

As metabolomics assessments were conducted simultaneously for cases and controls in both cohorts, we performed a pooled analysis. Metabolites were naturally log-transformed and auto-scaled to approximate normal distribution before formal analysis.

Covariate data obtained in each cohort were harmonized. The characteristics between lung cancer cases and matched controls were compared using Student’s *t*-test for continuous variables and Pearson’s chi-squared test for categorical variables. For the primary pooled analysis, we applied conditional logistic regression to estimate the odds ratio (OR) and 95% confidence interval (CI) per one standard deviation increase in the naturally log-transformed level of each known metabolite with lung cancer risk. The statistical models were conditioned on the matching variables and controlled for the body mass index (BMI) group (underweight: < 18.5 kg/m^2^, normal weight: 18.5–25 kg/m^2^, overweight: 25–30 kg/m^2^, obese: ≥ 30 kg/m^2^), hours since last meal (continuous; to account for length of fasting), physical activity (continuous; hours/week), fruits and vegetables consumption (continuous; servings/week), smoking status (categorical: never, former, current, and unknown), and hormone use (categorical: not a current user, current user, not applicable, unknown). Physical activity estimates the average total hours per week of walking or exercise in the CPS-II Nutrition cohort, while it estimates the average hours per day during the past 2 years in the CPS-3 cohort. We harmonized the variables and converted them into hours per week. The covariates were selected based on the literature review and a Directed Acyclic Graph. We removed the observations with any missing data for continuous covariates. We assigned an unknown category for missing data for categorical covariates. If a case was removed, its matching control was removed simultaneously, and vice versa. For the primary pooled analysis, 116 case–control pairs were removed due to missing values in hours since the last meal, physical activity, and fruit and vegetable consumption for either case or its matching control (Additional file 1: Fig. S1). Benjamini–Hochberg approach was used to calculate false discovery rates (FDRs) to correct for multiple comparisons. Metabolites associated with lung cancer risk at FDR < 0.2 were deemed statistically significant. To gain more biological responses of lung cancer, we focused on metabolites associated with lung cancer risk at *P* < 0.05 (*P*-value from statistical models before multiple comparison corrections) and further described and summarized the pathways in which these metabolites were involved.

We conducted an agglomerative hierarchical clustering analysis to group the lung cancer-associated metabolites (*P* < 0.05) based on their similarities. Pearson correlation was calculated between each pair of metabolites and then used for distance measure. Euclidean distance was computed between each pair of metabolites and returned the distance matrix. We then used the Ward clustering method to compute the similarity of the two clusters for merging [26]. The R package “*pheatmap*” was used for this analysis and result visualization.

We further examined the associations stratified by sex and by years between blood draw and lung cancer diagnosis (< 3 years, ≥ 3 years) using conditional logistic regression with the same set of covariates but excluding hormone use for males. The goal of the stratified analysis by years since the blood draw was to identify metabolites that may potentially serve as early detection biomarkers of lung cancer. Additionally, we stratified the analysis by smoking status (never, ever), by stage (localized, regional, distant), and by histological subtypes (squamous cell carcinoma, adenocarcinoma) using unconditional logistic regression, adjusting for matching variables as well as BMI group, hours since last meal, physical activity, fruits and vegetables consumption, hormone use, and smoking status (only for stage- and subtype-stratified analyses). For stratified analyses by smoking status, stage, and subtype where the unconditional logistic regression was applied, matching factors were adjusted as covariates in the model. If a case was removed due to missing covariates, its matching control would not be removed if the control has completed the covariates information, and vice versa. The lung cancer stage was examined according to the Surveillance, Epidemiology and End Results (SEER) stage at diagnosis: localized (invasive tumors confined to the lung); regional (tumors that extend to adjacent tissue or regional lymph nodes); distant (tumors are metastasized). The lung cancer histological subtype was categorized using ICD-O-3 morphology codes [27]. The morphology codes for each subtype can be found in supplementary materials. *P* for interaction was calculated using the likelihood ratio test. To test heterogeneity by stage and by subtype, we used the “*eh_test_subtype*” function in the R package “*riskclustr*” [28]. This function is designed for the test of etiologic heterogeneity across disease subtypes in the context of the case–control study. *P*-heterogeneity < 0.05 was considered statistically significant.

To examine the robustness of the results, we conducted a series of sensitivity analyses: (1) we recategorized the smoking variables into 7 categories (never, current smoker for < 50 years, current smoker for ≥ 50 years, former smoker quit < 10 years ago, former smoker quit 10–20 years ago, former smoker quit ≥ 20 years ago, unknown) based on smoking status and duration of time and adjusted it in the main analysis; (2) we further adjusted for cohort (CPS-II Nutrition, CPS-3) in the analysis to evaluate if any differences between cohorts (e.g., age of blood samples) would affect the main analysis results; (3) we also examined the associations stratified by years between blood draw and lung cancer diagnosis (< 5 years, ≥ 5 years).

All analyses were conducted using R (version 4.1.0.).

## Results

### Population characteristics

A total of 623 case–control pairs with an average age of 66.9 (± 8.6) years at blood draw were included in the analysis. Among the 1246 participants, 52.5% were female and the majority (96.1% in cases and 96.6% in controls) were white. Compared with controls, the average hours since the last meal for lung cancer cases was smaller. Additionally, cases were more likely to be current and former smokers (Table 1). Lung cancer cases were on average diagnosed at an age of 72.9 (± 10.3) years and the median time between blood draw and lung cancer diagnosis was 5.0 years (IQR: 7.0 years). Among cases, 46.5% were at a distant stage and 50.1% were adenocarcinoma.

**Table 1:** Participant characteristics of a nested, matched ^a^ case–control study in the Cancer Prevention Study-II (CPS-II) Nutrition and CPS-3 Cohort (for primary pooled analysis)

	**Case** **(*n* = 623)**	**Control (*n* = 623)**	***P*-value **^**b**^
**Age at blood draw (years), mean (SD)**	66.9 (8.57)	66.9 (8.57)	Matched
**Sex, *n* (%)**			Matched
Male	296 (47.5)	296 (47.5)	
Female	327 (52.5)	327 (52.5)	
**Race, *n* (%)**			Matched^**d**^
White	599 (96.1)	602 (96.6)	
Black	8 (1.3)	8 (1.3)	
Other/unknown	16 (2.6)	13 (2.1)	
**Body mass index group, *n* (%)**			0.09
< 18.5 kg/m^2^	10 (1.6)	7 (1.1)	
18.5–25 kg/m^2^	235 (37.7)	274 (44.0)	
25–30 kg/m^2^	245 (39.3)	235 (37.7)	
≥ 30 kg/m^2^	133 (21.3)	107 (17.2)	
**Hours since last meal, mean (SD)**	2.12 (2.06)	2.40 (2.26)	0.02
**Physical activity (hours/week), mean (SD)**	3.20 (7.08)	3.81 (8.48)	0.16
**Fruits and vegetables consumption (servings/week), mean (SD)**	30.0 (16.0)	31.7 (16.7)	0.06
**Smoking status, *n* (%)**			< 0.01
Never	114 (18.3)	318 (51.0)	
Former	387 (62.1)	275 (44.1)	
Current	114 (18.3)	15 (2.4)	
Unknown	8 (1.3)	15 (2.4)	
**Hormone use, *n* (%)**			0.96
Not a current user	192 (30.8)	199 (31.9)	
Current user	117 (18.8)	111 (17.8)	
Not applicable ^c^	296 (47.5)	296 (47.5)	
Unknown	18 (2.9)	17 (2.7)	
**Age at diagnosis (years), mean (SD)**	72.9 (10.3)	NA	NA
**Stage **^**e**^**, *n* (%)**			NA
Localized	150 (24.1)	NA	
Regional	151 (24.2)		
Distant	290 (46.5)		
Unknown	32 (5.1)		
**Subtype **^**f**^**, *n* (%)**			NA
Squamous cell carcinoma	97 (15.6)	NA	
Small cell carcinoma	54 (8.7)		
Adenocarcinoma	312 (50.1)		
Large cell carcinoma	15 (2.4)		
Non-small cell carcinoma	70 (11.2)		
Other carcinoma	75 (12.0)		

*NA* Not applicable

^a^ Controls were matched to cases by age at blood draw (± 6 months), sex, race, and date of blood draw (± 30 days). Seven matched controls became cases later. ^b^* P-*Values were obtained from independent *t*-tests (continuous) or chi-square tests (categorical). ^c^ This category were all males. ^d^ Three lung cancer cases from CPS-II Nutrition cohort did not match on the controls on race, so three other/unknown race cases were matched to white controls. All other matching criteria were satisfied. ^e^ The lung cancer stage was examined according to the Surveillance, Epidemiology and End Results (SEER) stage at diagnosis. Localized: invasive tumors confined to the lung; regional: tumors that extend to adjacent tissue or regional lymph nodes; distant: tumors are metastasized. ^f^ The lung cancer subtype was categorized using ICD-O-3 morphology code. The details can be found in supplementary materials

### Sixty-two metabolites were associated with lung *cancer* risk, mainly in lipid and amino acid metabolism

**Fig. 1:**
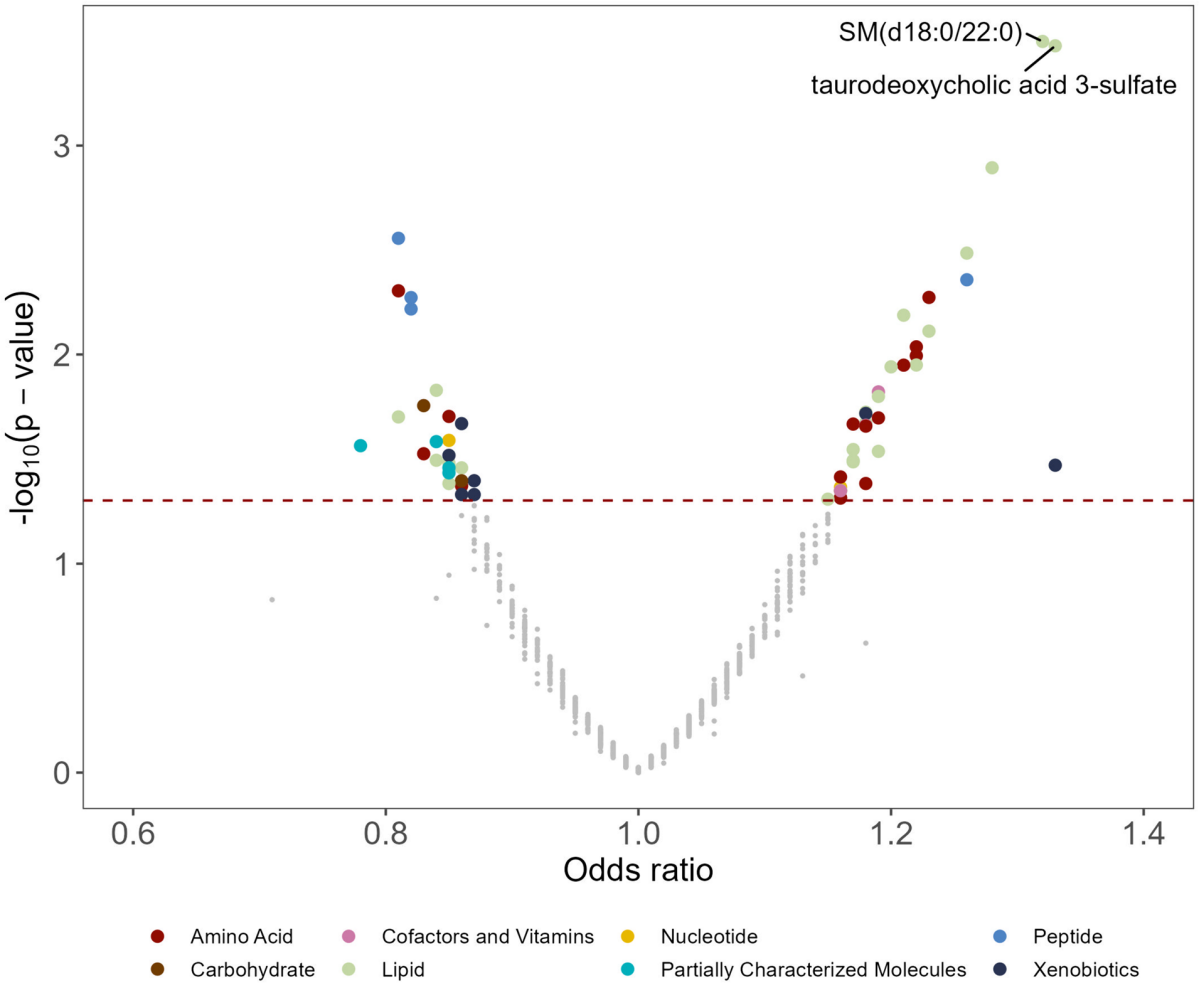
A volcano plot of associations between metabolites and lung cancer risk in the entire population. The *X*-axis denotes the odds ratio of lung cancer-metabolite associations. Odds ratios (95% confidence intervals) per one standard deviation increase in natural log-transformed level of each known metabolite with lung cancer risk were estimated from conditional logistic regression models, matched on age at blood draw, sex, race, and date of blood draw. Models were adjusted for body mass index group (underweight, healthy weight, overweight, obesity), hours since last meal (continuous), physical activity (continuous, hours/week), fruits and vegetables consumption (continuous, servings/week), smoking status (never, former, current, unknown), hormone use (not a current user, current user, not applicable, unknown). The *Y*-axis denotes the negative log_10_ of the *P*-value in the lung cancer-metabolite association. Different colors were used to represent different pathways where the metabolites are involved. The dark red dashed line represents *P*-value = 0.05. SM (d18:0/22:0) and taurodeoxycholic acid 3-sulfate were associated with lung cancer risk (FDR < 0.2). SM (d18:0/22:0), behenoyl dihydrosphingomyelin (d18:0/22:0)

**Fig. 2:**
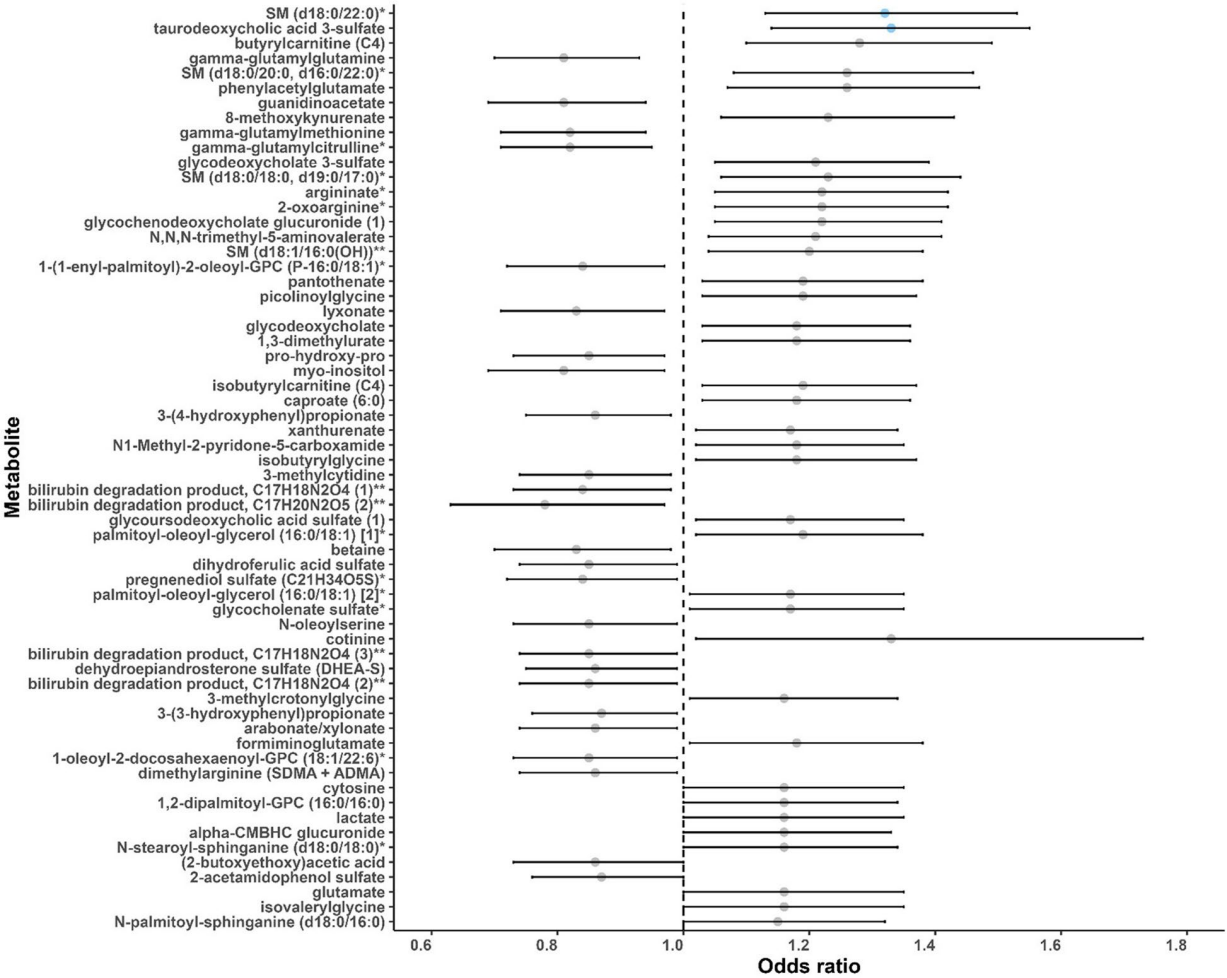
A forest plot of associations between metabolites and lung cancer risk (*P* < 0.05) in the entire population. Odds ratios (95% confidence intervals) per one standard deviation increase in natural log-transformed level of each known metabolite with lung cancer risk were estimated from conditional logistic regression models, matched on age at blood draw, sex, race, and date of blood draw. Models were adjusted for body mass index group (underweight, healthy weight, overweight, obesity), hours since last meal (continuous), physical activity (continuous, hours/week), fruits and vegetables consumption (continuous, servings/week), smoking status (never, former, current, unknown), hormone use (not a current user, current user, not applicable, unknown). Each dot represents the odds ratio of the association, with the whiskers representing the 95% confidence interval. The dots are arranged in ascending order based on the *P*-values of the associations, starting from the smallest *P* to the largest. Blue dots represent the metabolites associated with lung cancer risk at FDR < 0.2. The dashed vertical line represents the odds ratio of one. SM (d18:0/22:0), behenoyl dihydrosphingomyelin (d18:0/22:0); SM (d18:0/20:0, d16:0/22:0), sphingomyelin (d18:0/20:0, d16:0/22:0); SM (d18:0/18:0, d19:0/17:0), sphingomyelin (d18:0/18:0, d19:0/17:0); SM (d18:1/16:0 (OH)), hydroxypalmitoyl sphingomyelin (d18:1/16:0(OH)). * Putative identifications that are not confirmed with a purified standard (not tier 1). ** Putative identifications for which a standard is not available (not tier 1). Metabolites that are structurally similar but have a side group that could not be placed definitively in the molecule were given the same chemical name followed by a number in parentheses to differentiate them from each other

**Fig. 3:**
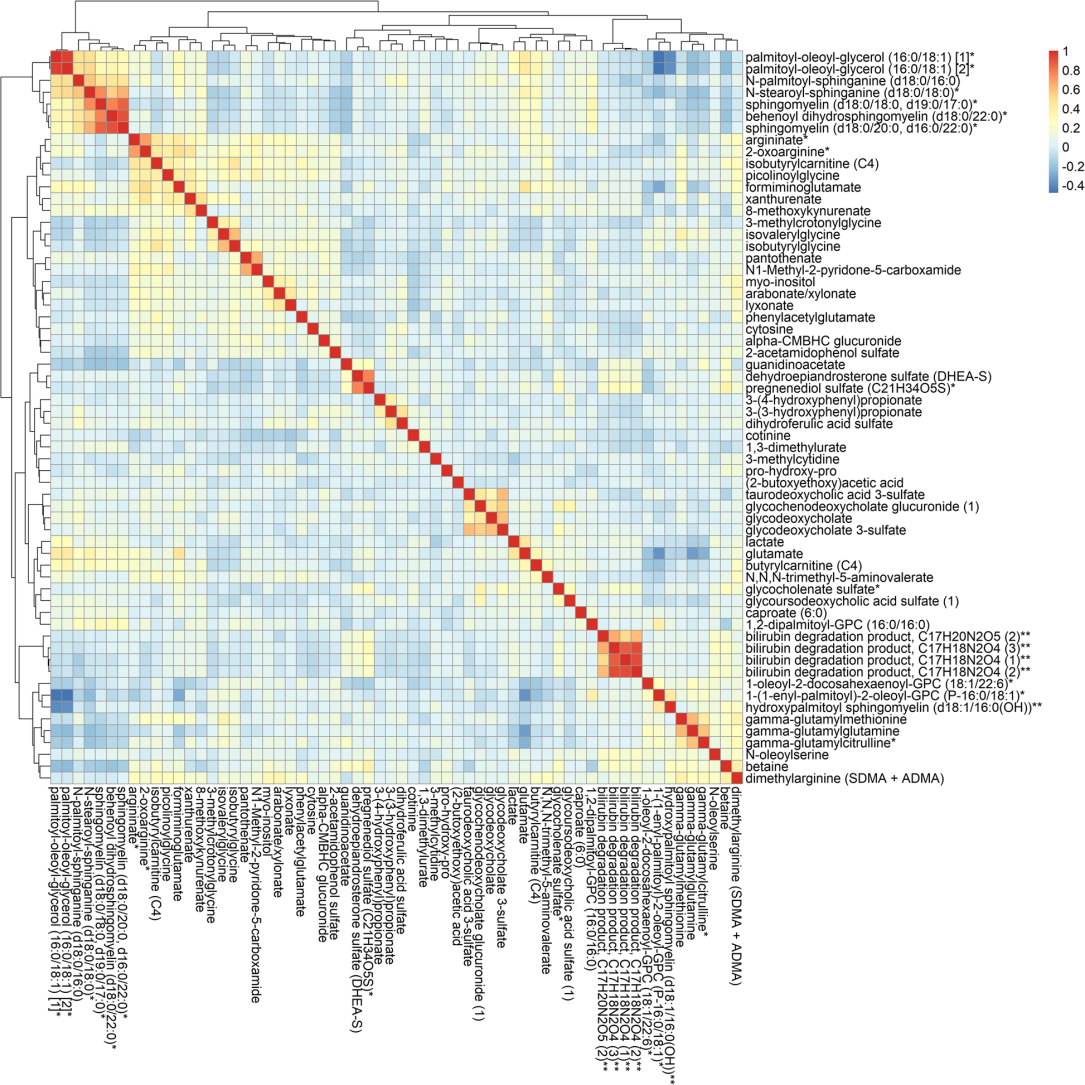
Agglomerative hierarchical clustering heatmap of the Pearson’s correlation coefficients among the sixty-two metabolites associated with lung cancer risk (*P-*value < 0.05). * Putative identifications that are not confirmed with a purified standard (not tier 1). ** Putative identifications for which a standard is not available (not tier 1). Metabolites that are structurally similar but have a side group that could not be placed definitively in the molecule were given the same chemical name followed by a number in parentheses to differentiate them from each other

In the main analysis, two metabolites were significantly associated with lung cancer risk: sphingomyelin (SM) (d18:0/22:0) (OR: 1.32, 95% CI: 1.13, 1.53; FDR = 0.15) and taurodeoxycholic acid 3-sulfate (OR: 1.33, 95% CI: 1.14, 1.55; FDR = 0.15) (Figs. 1 and 2, Additional file 2: Table S1). A total of 62 metabolites were associated with lung cancer risk at *P* < 0.05 (Fig. 2). Among the 62 metabolites, 37 metabolites showed positive associations (OR range: 1.15–1.33) and 25 had negative associations (OR range: 0.78–0.87) with lung cancer risk. Agglomerative hierarchical clustering analysis among the 62 metabolites revealed that an additional 2 SMs and 1 dihydroceramide are moderately to highly correlated with SM (d18:0/22:0) (Fig. 3). These metabolites were characterized mainly as lipids (39%), amino acids (24%), and xenobiotics (11%). (Additional file 1: Fig. S2). The lipid metabolism can include seven categories including sphingolipids, bile acids, phospholipids, fatty acids, glycerolipids, steroids, and eicosanoids. Specifically, higher levels of the metabolites identified in sphingolipid metabolism (OR range: 1.15–1.32), bile acid metabolism (OR range: 1.17–1.33), and fatty acid metabolism (OR range: 1.18–1.28) were associated with a higher risk of developing lung cancer (Additional file 2: Table S2). For the metabolites in sphingolipid metabolism, three were dihydrophingomyelins, two were dihydroceramides, and one was sphingomyelin. For metabolites in bile acid metabolism, one belonged to primary bile acid metabolism, while the other five belonged to secondary bile acid metabolism. The amino acids metabolism mainly contains arginine and proline metabolism, branched-chain amino acid metabolism, and aromatic amino acid metabolism. Likewise, higher levels of the metabolites identified in branched-chain amino acid metabolism (OR range: 1.16–1.19) were associated with a higher risk of developing lung cancer (Additional file 2: Table S3).

### SM (d18:0/22:0) and taurodeoxycholic acid 3-sulfate were consistently positively associated with lung *cancer* risk across strata

SM (d18:0/22:0) was consistently positively associated with lung cancer risk across strata, though the associations in some strata were not statistically significant (*P* < 0.05). When stratified by sex, SM (d18:0/22:0) was associated with (*P* < 0.05) higher lung cancer risk in both men and women (*P*-heterogeneity = 0.50) (Table 2). Notably, among cases diagnosed within 3 years of blood draw (*n* = 177), one standard deviation increase in natural log-transformed SM (d18:0/22:0) levels was associated with 55% higher risk of lung cancer (OR: 1.55, 95% CI: 1.12, 2.13), while the same amount of increase was associated with 26% higher risk among cases diagnosed beyond 3 years after blood draw (OR: 1.26, 95% CI: 1.06, 1.50) (*n* = 446), compared to matched controls. However, the association of SM (d18:0/22:0) with lung cancer risk did not differ by follow-up time (*P*-heterogeneity = 0.33). When stratified by smoking status, SM (d18:0/22:0) was associated with higher lung cancer risk among ever-smokers (*P* = 0.02). The association was also positive, albeit non-significant, among never-smokers (*P* = 0.61). There was no interaction between SM (d18:0/22:0) and smoking status (*P*-heterogeneity = 0.49). No heterogeneity was observed when stratified SM (d18:0/22:0) associations by lung cancer stage (*P*-heterogeneity = 0.77) and subtype (*P*-heterogeneity = 0.12).

**Table 2:** Associations between sphingomyelin (d18:0/22:0), taurodeoxycholic acid 3-sulfate and lung cancer stratified by sex, by follow-up time, by smoking status, by stage, and by subtype

Strata	Number of lung cancer cases	Number of controls	OR (95%CI)	*P*-value	*P-*heterogeneity^c^
**Sphingomyelin (d18:0/22:0)**					
Overall ^a^	623	623	1.32 (1.15, 1.53)	< 0.001^d^	NA
Sex ^a^					0.50
Male	296	296	1.27 (1.01, 1.60)	0.039	
Female	327	327	1.35 (1.10, 1.65)	0.004	
Follow-up time ^a^					0.33
< 3 years	177	177	1.55 (1.12, 2.13)	0.008	
≥ 3 years	446	446	1.26 (1.06, 1.50)	0.008	
Smoking status ^b^					0.49
Never-smoker	118	348	1.07 (0.83, 1.36)	0.609	
Ever-smoker	539	300	1.21 (1.04, 1.42)	0.017	
Stage ^b^					0.77
Localized	158	663	1.15 (0.94, 1.41)	0.179	
Regional	162	663	1.24 (1.01, 1.53)	0.040	
Distant	311	663	1.13 (0.96, 1.32)	0.144	
Subtype^b^					0.12
Squamous cell carcinoma	102	663	1.30 (1.01, 1.68)	0.043	
Adenocarcinoma	334	663	1.09 (0.93, 1.27)	0.293	
**Taurodeoxycholic acid 3-sulfate**					
Overall ^a^	623	623	1.33 (1.14, 1.55)	< 0.001^d^	NA
Sex ^a^					0.91
Male	296	296	1.34 (1.06, 1.68)	0.013	
Female	327	327	1.31 (1.05, 1.63)	0.017	
Follow-up time ^a^					0.50
< 3 years	177	177	1.48 (1.08, 2.03)	0.015	
≥ 3 years	446	446	1.28 (1.07, 1.53)	0.008	
Smoking status ^b^					0.62
Never-smoker	118	348	1.09 (0.87, 1.38)	0.449	
Ever-smoker	539	300	1.16 (1.00, 1.34)	0.053	
Stage ^b^					0.08
Localized	158	663	1.22 (1.00, 1.49)	0.045	
Regional	162	663	1.22 (1.00, 1.50)	0.052	
Distant	311	663	1.09 (0.93, 1.28)	0.266	
Subtype^b^					0.29
Squamous cell carcinoma	102	663	1.14 (0.89, 1.46)	0.308	
Adenocarcinoma	334	663	1.03 (0.99, 1.19)	0.729	

*OR* Odds ratio, *CI* Confidence interval, *NA* Not applicable

^a^ ORs (95% CI) were estimated from conditional logistic regression models, conditioned on age at blood draw, sex, race, and date of blood draw and adjusted for body mass index group, hours since last meal, physical activity, fruits and vegetables consumption, smoking status, and hormone use (for overall population and females). ^b^ ORs (95% CI) were estimated from unconditional logistic regression models, adjusted for age at blood draw, sex, race, date of blood draw, body mass index group, hours since last meal, physical activity, fruits, and vegetables consumption, hormone use, and smoking status (only for stage-stratified and subtype-stratified analyses). The lung cancer stage was examined according to the Surveillance, Epidemiology and End Results (SEER) stage at diagnosis. Localized: invasive tumors confined to the lung; regional: tumors that extend to adjacent tissue or regional lymph nodes; distant: tumors are metastasized. The lung cancer subtype was categorized using ICD-O-3 morphology code. The details can be found in supplementary materials. ^c^ For stratified analysis by sex, follow-up time, and smoking status, likelihood ratio test was used to test the interaction; for lung cancer stage and subtype, we used the “*eh_test_subtype*” function in R package “*riskclustr*”. The model was adjusted for age at blood draw, sex, race, date of blood draw, body mass index group, hours since last meal, physical activity, fruits and vegetables consumption, and smoking status. We took off the hormone use variable here, given that the model could not converge due to the collinearity between sex and hormone use. ^d^ FDR = 0.148

Taurodeoxycholic acid 3-sulfate was associated with higher lung cancer risk in male, female, cases diagnosed within and beyond 3 years of blood draw, and those at localized stage (*P* < 0.05) (Table 2). Likewise, among cases diagnosed within 3 years of blood draw (*n* = 177), one standard deviation increase in natural log-transformed taurodeoxycholic acid 3-sulfate levels was associated with 48% higher risk of lung cancer (OR: 1.48, 95% CI: 1.08, 2.03), while the same amount of increase was associated with 28% higher risk among cases diagnosed beyond 3 years after blood draw (OR: 1.28, 95% CI: 1.07, 1.53) (*n* = 446), compared to matched controls. No heterogeneity was observed when stratified taurodeoxycholic acid 3-sulfate associations by sex (*P*-heterogeneity = 0.91), follow-up time (*P*-heterogeneity = 0.50), smoking status (*P*-heterogeneity = 0.62), lung cancer stage (*P*-heterogeneity = 0.08), and subtype (*P*-heterogeneity = 0.29).

### Lung *cancer*-associated metabolic profiles varied between ever- and never-smokers

**Fig. 4:**
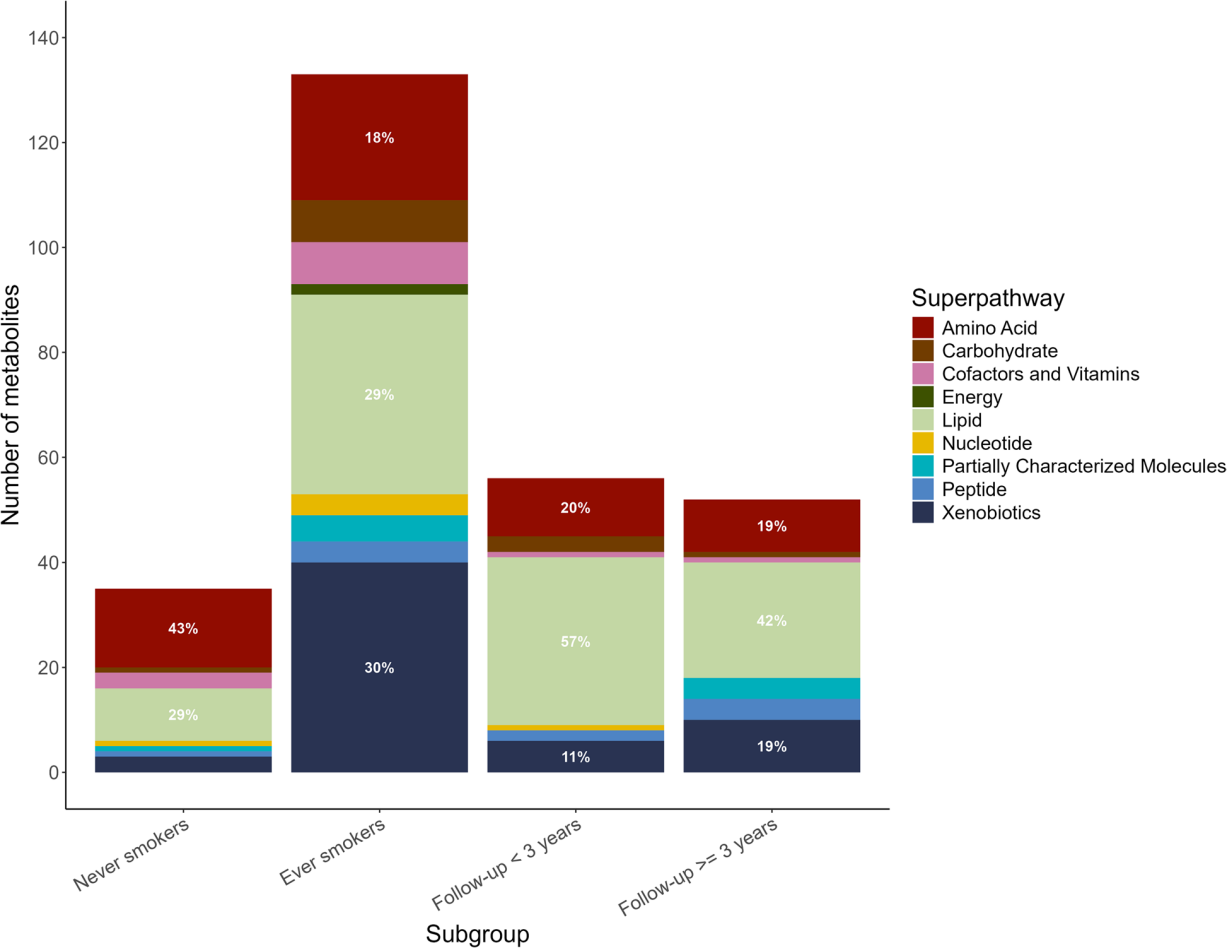
Descriptive distribution of metabolic pathways that contain the lung cancer-associated metabolites at *P-*value < 0.05 by smoking status and follow-up time

We observed that the distribution of metabolic pathways containing lung cancer-associated metabolites (*P* < 0.05) varied by smoking status, sex, tumor stage, and histological subtypes (Additional file 1: Fig. S3). Results for stratified analyses can be found in supplementary materials (Additional file 2: Table S4–S14). We identified 65 metabolites associated with lung cancer risk (FDR < 0.2) in ever-smokers (Additional file 1: Fig. S4–S5, Additional file 2: Table S9), while none in never-smokers (Additional file 2: Table S8). Interestingly, the four most significant metabolites in ever-smokers were tobacco metabolites, which were cotinine, hydroxycotinine, cotinine N-oxide, and 3-hydroxycotinine glucuronide. Looking closely at the pathways where metabolites associated with lung cancer risk (*P* < 0.05) were involved, there were greater proportion of metabolites in xenobiotic- and lipid-associated metabolic pathways in ever-smokers compared to never-smokers (Fig. 4). However, the amino acid- and lipid-associated metabolic pathways were more pronounced in never-smokers. As for stratified analysis by follow-up time, the proportion of lipid- and amino acid-associated metabolic pathways were similar (Fig. 4), but lung cancer-associated metabolites (*P* < 0.05) were largely different (Additional file 2: Table S6–S7). A more distinct perturbation of metabolites in lipid pathways was observed in female cases than in male cases, those at regional and distant stages than those at a localized stage, adenocarcinoma cases than squamous cell carcinoma cases (Additional file 1: Fig. S3). A more distinct perturbation of metabolites in amino acids pathways was observed in cases at localized stages than those at other stages. For subtype-stratified analysis, we identified 12 metabolites significantly associated with lung cancer risk (FDR < 0.2) in squamous cell carcinoma (Additional file 2: Table S13), while one in adenocarcinoma (Additional file 2: Table S14). Notably, lipid and amino acid metabolism are major metabolic pathways involved in lung cancer development, accounting for 47% to 80% of all lung cancer-associated metabolites at *P* < 0.05, either among all participants or in subgroup analyses (Fig. 4, Additional file 1: Fig. S2–S3).

### Sensitivity analyses

Sensitivity analyses revealed that 63% of lung cancer-associated metabolites (*P <* 0.05) remained when replacing four-category smoking variables with seven-category smoking variables that further included smoking duration in the model. The associations of SM (d18:0/22:0), taurodeoxycholic acid 3-sulfate, and lung cancer risk in cases diagnosed within 5 years of blood draw remained significant (OR: 1.47, 95% CI: 1.15, 1.89, *P* = 0.003 and OR: 1.60, 95% CI: 1.25, 2.07, *P* < 0.001, respectively). The number and identities of metabolites (*P* < 0.05) and their corresponding ORs from models were nearly the same before and after including the cohort variable in the model, which indicates that the effects of any differences between cohorts were too small to detect (results not shown).

## Discussion

In this large pooled analysis of prospective cohort studies on examining metabolic profiles in association with lung cancer risk using untargeted metabolomics, SM (d18:0/22:0), a sphingolipid, and taurodeoxycholic acid 3-sulfate, a bile salt, were positively associated with lung cancer risk regardless of smoking status, follow-up time, sex, stage, and subtype, though the associations in some strata did not survive *P* < 0.05. Lipid (sphingolipid, bile acid, phospholipids, and fatty acids pathways) and amino acid metabolism (arginine and proline metabolism, branched-chain amino acids, and aromatic amino acids) may play an important role in lung cancer etiology. Distinct metabolic profiles between never and ever-smokers suggest heterogeneity in lung cancer etiology by smoking status.

Lipid metabolism has been associated with the initiation and progression of lung cancer [29]. Consistently, we observed an extensive perturbation of metabolites in lipid pathways in our study. Sphingolipids are ubiquitous bioactive components of cell membranes and also play an important role in cell signaling in various physiological processes [30–32]. Previous studies have ranked sphingolipid metabolism as one of the top dysregulated pathways in lung cancer development in human studies [33, 34]. In particular, several key sphingolipids (e.g., sphingosine-1-phosphate (S1P), ceremide) and related enzymes (e.g., sphingosine kinases (SphK1/2), ceramide kinases (Cerk)) were found to play crucial roles in lung cancer etiology by disrupting universe cellular processes, regulating downstream signaling pathways, and affecting tumor microenvironment [32, 35–39]. In our study, higher levels of several sphingolipids were associated with a higher risk of lung cancer, suggesting the aberrantly active activity of sphingolipids in lung cancer development. Upregulation of these metabolites, as precursors of ceramide, may be an indicator of increased synthesis of ceramide/S1P or abnormal ceramide-to-S1P ratio. Specifically, the imbalance of ceramide/S1P has been suggested to be associated with unrelenting airway inflammation which could ultimately cause increased oxidative stress and aberrant signaling [40, 41], increased apoptosis and senescence [42–44], impaired immunity [45, 46], lung remodeling [47, 48], increased lung permeability, and altered surfactant [49].

Perturbation of bile acid metabolism in lung cancer cases also warrants attention. Bile acids are known for the promotion of the absorption of lipids, and they also play an important role in cell signaling and maintaining human body homeostasis. Recent studies have characterized the role of bile acids in cancer development and progression, albeit the research is in its infancy [50–52]. In our study, we identified one conjugated primary bile acid and five conjugated secondary bile acids and their derivatives, which were all positively associated with lung cancer risk. Consistently, another study reported much higher serum-free secondary bile acids (deoxycholic acid and ursodeoxycholic acid) and primary bile acid (chenodeoxycholic acid) in non-small cell lung cancer (NSCLC) patients than the healthy controls [52]. Due to the close link between bile acids and microbes in the gut [50, 53, 54], higher expression of secondary bile acids identified in the current study may be an indicator of the abnormal structure of microbial communities. However, details remain unclear on how bile acid metabolism is regulated in lung cancer. Further investigations on bile acid metabolism and the interaction between secondary bile acids and gut microbiota in lung cancer etiology are needed.

Particularly, we observed higher levels of SM (d18:0/22:0), a sphingolipid, was consistently associated with lung cancer risk among all participants (FDR < 0.2) and across different strata (*P* < 0.05). SM (d18:0/22:0) is involved in the dihydrosphingomyelins pathway. Additionally, we observed higher levels of taurodeoxycholic acid 3-sulfate, a bile salt, was associated with higher lung cancer risk in the entire population (FDR < 0.2) and several subgroups (male, female, cases diagnosed within and beyond 3 years of blood draw, and those at localized stage) (*P* < 0.05). Taurodeoxycholic acid 3-sulfate is involved in secondary bile acid metabolism. Notably, the association of SM (d18:0/22:0) and taurodeoxycholic acid 3-sulfate with lung cancer was the strongest among cases diagnosed within 3 years of follow-up, but the association was still significant though weaker among cases diagnosed beyond 3 years of follow-up, which shows their great potential as an early detection and possibly a screening biomarker for lung cancer. In addition, we identified three additional SMs positively associated with lung cancer risk before correcting for multiple comparisons, including SM (d18:1/16:0 (OH)), SM (d18:0/18:0, d19:0/17:0), and SM (d18:0/20:0, d16:0/22:0). Previous studies have shown the changes of SMs alone or in combination with other molecules can predict the recurrence of specific types of lung cancer [55, 56] and can differentiate early-stage lung cancer from controls [57]. Additionally, our study replicated several metabolites previously found to be associated with lung cancer risk, including cotinine, lactate, and glutamate [14]. Increased plasma cotinine levels were associated with a 33% higher risk of lung cancer in the present study, which is consistent with previous findings [14, 58, 59].

In addition, we observed a certain degree of perturbation of amino acids metabolism in lung cancer cases compared to matched controls, including arginine and proline metabolism (arginine and proline metabolism, creatine metabolism), branched-chain amino acids metabolism (leucine, isoleucine, and valine metabolism), and aromatic amino acids metabolism (tryptophan metabolism). Amino acid metabolism plays a crucial role in various cellular processes including protein synthesis and energy production, which was found involved in tumor development and progression. More specifically, arginine and proline metabolism plays an important role in metabolic reprogramming in cancer [60, 61]. An increase in branched-chain amino acids (BCAAs) metabolism was thought to provide energy sources and contribute to tumor growth [62]. Tryptophan and its metabolites have been reported to be significantly involved in the immune escape of lung cancer, such as promoting immune suppression [63].

We observed distinct metabolic profiles associated with lung cancer risk by smoking status, suggesting the heterogeneity in lung cancer etiology between never-smokers and ever-smokers to a certain degree, though the detailed mechanisms were not clear. Specifically, we identified 65 metabolites associated with lung cancer risk (FDR < 0.2) in ever-smokers, while none in never-smokers. When considering lung cancer-associated metabolites at *P* < 0.05, we observed a more prominent perturbation of metabolites in xenobiotic-associated and lipid-associated pathways in ever-smokers compared to never-smokers. The SM(d18:0/22:0) association was stronger in ever-smokers than in never-smokers, suggesting that this pathway may be particularly relevant to lung cancers that develop as a result of cigarette smoking. Our findings provide extra evidence that lung cancer mechanisms may differ by smoking status. Consistent with previous findings, lung cancer in never-smokers and ever-smokers was suggested as two distinct disease processes, with different epidemiologic, clinical, and genetic characteristics [8, 10, 64–67]. Lung cancer-associated metabolites (*P* < 0.05) varied greatly between cases diagnosed within and beyond 3 years of blood draw, among different stages, as well as between squamous cell carcinoma and adenocarcinoma cases. These findings may suggest potential differences in metabolome associated with different rates of progression, stages, and subtypes. Limited studies have reported several metabolites in serum were differentially expressed in early stage versus advanced stage of lung cancer [68]. It is noteworthy that the number of cases is not very large in some strata in our analysis, which may lead to insufficient statistical power. Our findings should be validated by future studies. Overall, the perturbation of lipid levels was found to be a dominant characteristic across the entire study population, as well as in other subgroups, with the exception of lung cancer cases who were never-smokers, males, and at localized stage. A caveat is that the pathway differentiation by smoking status or by other strata was simply descriptive and did not involve statistical testing to determine the significance across the subgroups. We observed certain degrees of metabolites in xenobiotic-related pathways across the entire study population and subgroups, with the highest proportions in ever-smoking lung cancer cases (30%) followed by squamous cell carcinoma cases (27%). This may imply residual confounding arising from dietary factors as well as concurrent exposure to drugs and other chemical agents. Specifically, among ever-smoking lung cancer cases and squamous cell carcinoma cases, we observed ten and eight metabolites of caffeinated and decaffeinated coffee (e.g., caffeine, 1-methylurate, and 1,3-dimethylurate) [69], which were positively associated with the lung cancer risk. Previously, smoking has been associated with higher caffeine consumption [70, 71].

Previously, Seow et al. conducted a prospective nested case–control study with a focus on lung cancer-associated metabolic perturbation in urine samples collected from never-smoking Chinese women [21]. They found extensive urinal metabolic perturbation among lung cancer cases compared to controls, which suggests systematic changes in 1-carbon metabolism, oxidative stress and inflammation pathways, and nucleotide metabolism. Among never-smoking cases in our study, we did observe pathways related to 1-carbon metabolism, oxidative stress, and inflammation, including methionine, cysteine, and taurine metabolism, tocopherol metabolism, glutamate metabolism, and histidine metabolism. It is not reasonable to directly compare the results between our and Seow’s studies given differences in biosamples and metabolic profiling procedures, and heterogeneities in populations including races, ages, sex proportion, and dietary patterns.

To our knowledge, this is the largest prospective study of untargeted metabolomics on lung cancer risk. The current study has a large sample size, based upon the established cohort with well-characterized risk factors (e.g., detailed smoking histories, hormone use). We can perform stratified and in-depth analyses. Besides, pre-diagnosis blood samples provide valuable information on metabolic perturbations associated with lung cancer initiation and development, which is beneficial for early-detection biomarkers identification. In this study, we only included 887 known metabolites, with high levels of confidence in the annotation (Levels 1 and 2) [72] and high data quality, which makes our results more reliable compared to prior studies that reported all detected signals in the biosamples and claimed all the signals are unique compounds. Our study also has limitations. This analysis is based on one-time metabolic measurement, neglecting within-person variations over time. Thus, the dynamics of metabolites during lung cancer development were unknown. Additionally, Metabolic profiling using non-fasting blood samples, potentially introduced measurement errors in diet-related metabolites. Yet, the impact of fasting status was minimized by controlling for hours since the last meal in analyses. From the perspective of hypothesis generation, a loose threshold, *P* < 0.05, was used for gaining more information on biological pathways associated with lung cancer by smoking status. Simultaneously, the possibility of a false discovery rate increased [73]. It is noteworthy that the metabolome is sensitive and susceptible to influences from both endogenous and exogenous factors along with the computational nature of this study, caution is warranted in interpreting the results as the causality was not able to be established. Potential selection bias may exist, as population characteristics including sex, race, BMI, and smoking differed between the lung cancer cases included in the present analysis and those excluded due to unavailable blood samples. Also, our findings may lack generalizability to races other than white or younger populations.

## Conclusions

In this large pooled analysis of nested case–control studies of lung cancer metabolomics, we identified that pre-diagnosis changes in lipid metabolism and amino acid metabolism may play important roles in lung cancer etiology. Notably, SM (d18:0/22:0) and taurodeoxycholic acid 3-sulfate may be risk factors and potential screening biomarkers for lung cancer. Distinctive metabolic profiles by smoking status suggest heterogeneity in lung cancer etiology. Future studies are needed to validate our findings.

### Supplementary Information

Additional file 1: Figure S1. Flowchart of exclusion criteria for study participants in the primary pooled analysis. Figure S2. Distribution of super and sub pathways containing the sixty-two metabolites associated with lung cancer risk (*P*-value < 0.05). Figure S3. Descriptive distribution of metabolic pathways that contain the lung cancer-associated metabolites at *P*-value < 0.05 by sex, lung cancer stage, and subtype. Figure S4. Agglomerative hierarchical clustering heatmap of the Pearson’s correlation coefficients among the sixty-five metabolites associated with lung cancer risk in ever smokers (FDR < 0.2). Figure S5. A volcano plot of associations between metabolites and lung cancer risk in ever smokers.

Additional file 2: Table S1. Metabolites associated with lung cancer risk at *P*-value < 0.05 in the entire population. Table S2. Lipid-associated metabolic pathways components identified in lung cancer cases compared to controls. Table S3. Amino acids-associated metabolic pathways components identified in lung cancer cases compared to controls. Table S4. Metabolites associated with lung cancer risk at *P*-value < 0.05 in female stratum. Table S5. Metabolites associated with lung cancer risk at *P*-value < 0.05 in male stratum. Table S6. Metabolites associated with lung cancer risk at *P*-value < 0.05 in follow-up time ≤ 3 years stratum. Table S7. Metabolites associated with lung cancer risk at *P*-value < 0.05 in follow-up time > 3 years stratum. Table S8. Metabolites associated with lung cancer risk at *P*-value < 0.05 in never smokers stratum. Table S9. Metabolites associated with lung cancer risk at *P*-value < 0.05 in ever smokers stratum. Table S10. Metabolites associated with lung cancer risk at *P*-value < 0.05 in localized lung cancer stage stratum. Table S11. Metabolites associated with lung cancer risk at *P*-value < 0.05 in reginal lung cancer stage stratum. Table S12. Metabolites associated with lung cancer risk at *P*-value < 0.05 in distant lung cancer stage stratum. Table S13. Metabolites associated with lung cancer risk at *P*-value < 0.05 in squamous cell carcinoma stratum. Table S14. Metabolites associated with lung cancer risk at *P*-value < 0.05 in adenocarcinoma stratum.

## Data Availability

The datasets analyzed during the current study are not publicly available due to the privacy of individuals that participated in the study. The data will be shared on reasonable request to the corresponding author.

## Abbreviations

BCAAs: Branched-chain amino acids
BMI: Body mass index
Cerk: Ceramide kinases
CI: Confidence interval
CPS: Cancer Prevention Study
FDR: False discovery rates
ICC: Intraclass correlation coefficient
IQR: Interquartile range
NSCLC: Non-small cell lung cancer
OR: Odds ratio
S1P: Sphingosine-1-phosphate
SEER: Surveillance, Epidemiology and End Results
SM: Sphingomyelin
SphK1/2: Sphingosine kinases
UPLC-MS/MS: Ultrahigh-performance liquid chromatography-tandem mass spectrometry
