# Supplementary material for: Lung cancer metabolomics: a pooled analysis in the Cancer Prevention Studies
Source: BMC Med. 2024 Jun 24;22:262. doi: 10.1186/s12916-024-03473-1 (PMC11197282; doi:10.1186/s12916-024-03473-1)
Supplement: Supplementary file 1 — Additional file 1: Figure S1. Flowchart of exclusion criteria for study participants in the primary pooled analysis. Figure S2. Distribution of super and sub pathways containing the sixty-two metabolites associated with lung cancer risk (P-value < 0.05). Figure S3. Descriptive distribution of metabolic pathways that contain the lung cancer-associated metabolites at P-value < 0.05 by sex, lung cancer stage, and subtype. Figure S4. Agglomerative hierarchical clustering heatmap of the Pearson’s correlation coefficients among the sixty-five metabolites associated with lung cancer risk in ever smokers (FDR < 0.2). Figure S5. A volcano plot of associations between metabolites and lung cancer risk in ever smokers. [file 12916_2024_3473_MOESM1_ESM.docx]

**Supplemental Materials**

**Lung cancer metabolomics: A pooled analysis in the Cancer Prevention Studies**

Ziyin Tang^1^, Donghai Liang^1^*, Emily L. Deubler^2^, Jeremy A. Sarnat^1^, Sabrina S. Chow^1^, W. Ryan Diver^2-4^, Ying Wang^2^*

^1^ Gangarosa Department of Environmental Health, Rollins School of Public Health, Emory University, Atlanta, Georgia, United States.

^2^ Department of Population Science, American Cancer Society, Atlanta, Georgia, United States.

^3^ Barcelona Institute for Global Health (ISGlobal), Barcelona, Spain.

^4^ Universitat Pompeu Fabra (UPF), Barcelona, Spain.

*Corresponding authors:

Ying Wang, PhD

Senior Principal Scientist, Epidemiology Research

American Cancer Society, Inc.

3380 Chastain Meadows Pkwy NW Suite 200

Kennesaw, GA 30144

[ying.wang@cancer.org](mailto:ying.wang@cancer.org)

Donghai Liang, PhD

Assistant Professor, Gangarosa Department of Environmental Health

Rollins School of Public Health, Emory University

1518 Clifton Rd NE

Atlanta, GA, 30322

[donghai.liang@emory.edu](mailto:donghai.liang@emory.edu)

**Metabolic Profiling**

In contrast to targeted metabolomics focusing on specific group of metabolites, untargeted metabolomics profiling is a process that aims at comprehensive detection and relative quantification of small molecules in a sample, which provides valuable information on metabolic perturbations associated with lung cancer risk. Briefly, the plasma samples were treated with methanol to precipitate proteins and then centrifugated. Four sample fractions were dried and reconstituted in different solvent for measurement under four different platforms: 1) two fractions were analyzed by 2 separate reverse-phase (RP)/UPLC -MS/MS methods with positive-ion-mode electrospray ionization (ESI); 2) one fraction was analyzed by RP/UPLC-MS/MS with negative-ion-mode ESI; 3) one fraction was analyzed by hydrophilic interaction chromatography (HILIC)/UPLC-MS/MS with negative-ion-mode ESI. Lung cancer cases and matched controls were analyzed in the same batch in a blind manner to the lab technicians. We used 44 replicate samples from 34 CPS-II participants (24 duplicates and 10 triplicates) as quality control samples to assess intra- and inter-batch variation for calculating technical intraclass correlation coefficient (ICC). Individual metabolites were identified by comparison with Metabolon library consisting of > 5,400 commercially available purified standard compounds based on mass-to-charge ratio (m/z), retention index, and fragmentation. Peaks were quantified using area-under-the-curve. Day-to-day variation was corrected by setting median values for each compound to 1 for each run-day and each data point was normalized proportionately. Missing values were imputed to the observed minimum of the non-missing values.

**The Morphology Codes for Lung Cancer Subtype**

The morphology codes were: squamous cell carcinoma (8051-2, 8070-6, 8078, 8083-4, 8090, 8094, 8120, 8123), adenocarcinoma (8015, 8050, 8140-1, 8143-5, 8147, 8190, 8201, 8211, 8250-5, 8260, 8290, 8310, 8320, 8323, 8333, 8401, 8440, 8470-1, 8480-1, 8490, 8503, 8507, 8550, 8570-2, 8574, 8576), small cell carcinoma (8002, 8041-5), large cell carcinoma (8012-4, 8021, 8034, 8082), non-small cell carcinoma (8046), Other carcinoma (8003-4, 8022, 8030-3, 8035, 8200, 8240-1, 8243-6, 8249, 8430, 8525, 8560, 8562, 8575, 8010-1, 8020, 8230, 8000-1, 8800, 8801, 8980).


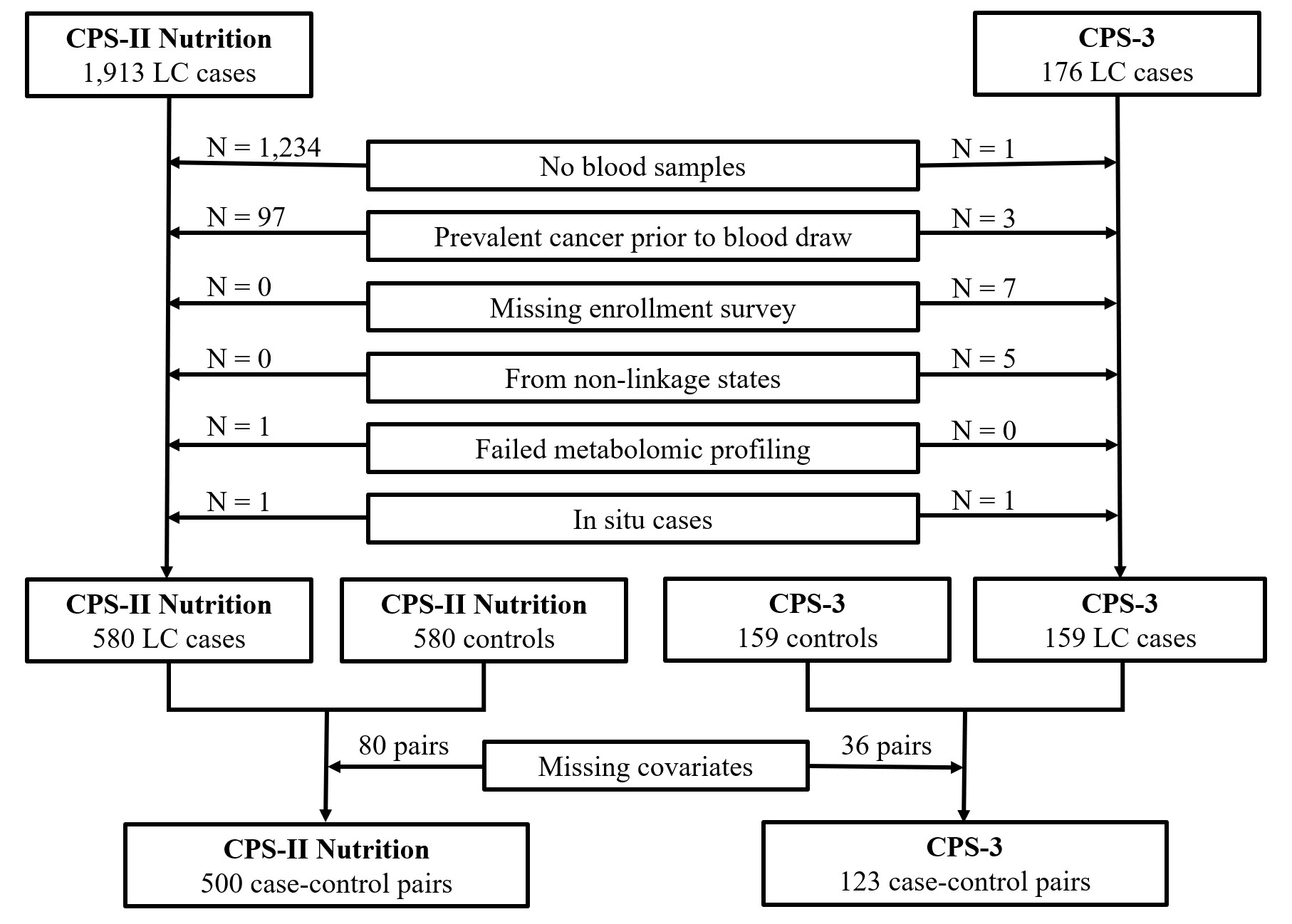


**Figure S1.** Flowchart of exclusion criteria for study participants in the primary pooled analysis.

Note: LC, lung cancer.

**
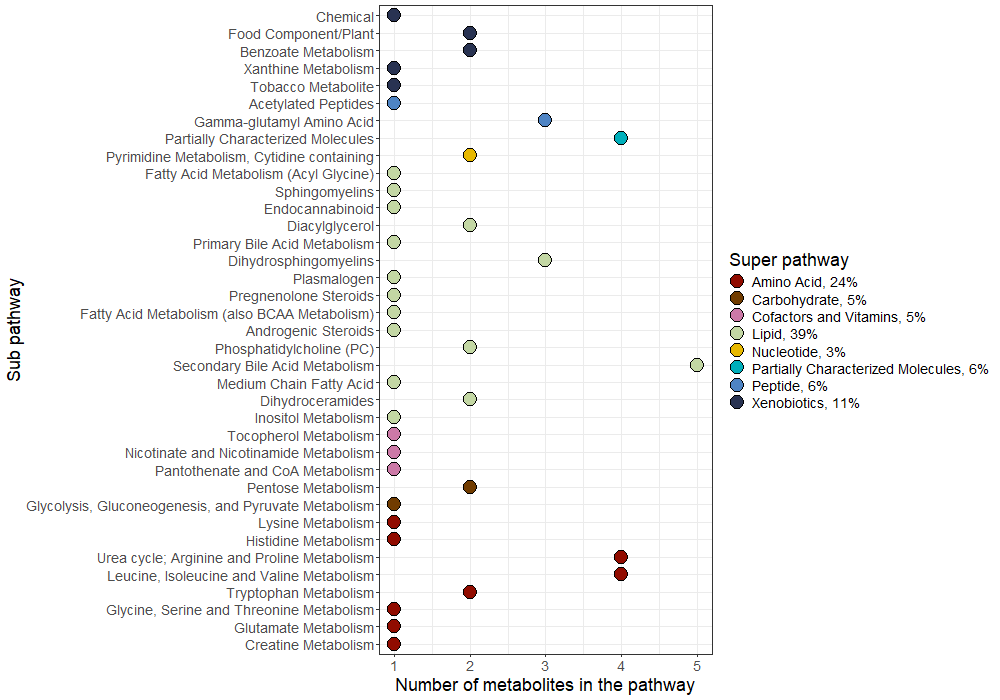
**

**Figure S2.** Distribution of super and sub pathways containing the sixty-two metabolites associated with lung cancer risk (*P-*value $<0.05$).


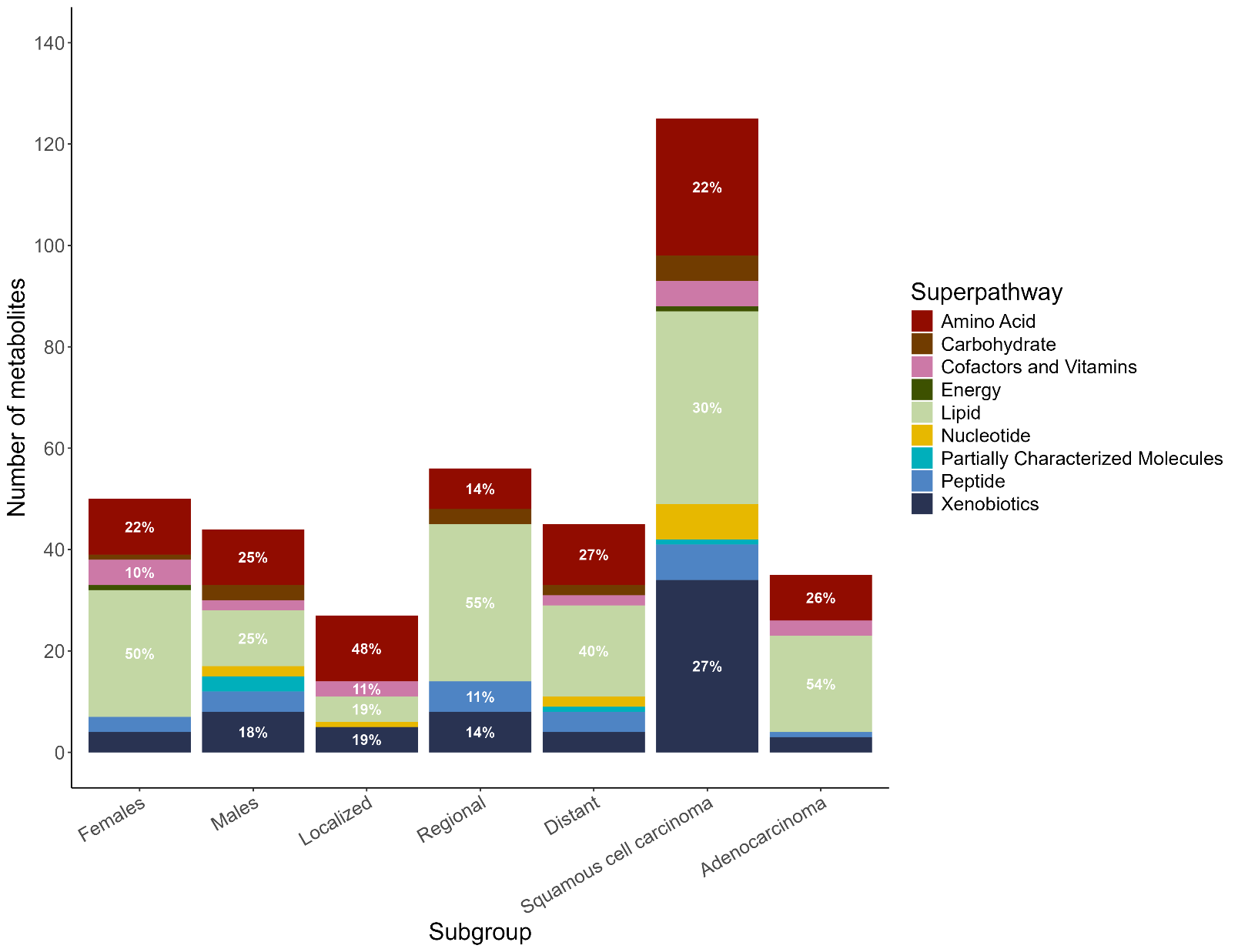


**Figure S3.** Descriptive distribution of metabolic pathways that contain the lung cancer-associated metabolites at *P-*value $<0.05$ by sex, lung cancer stage, and subtype.

**
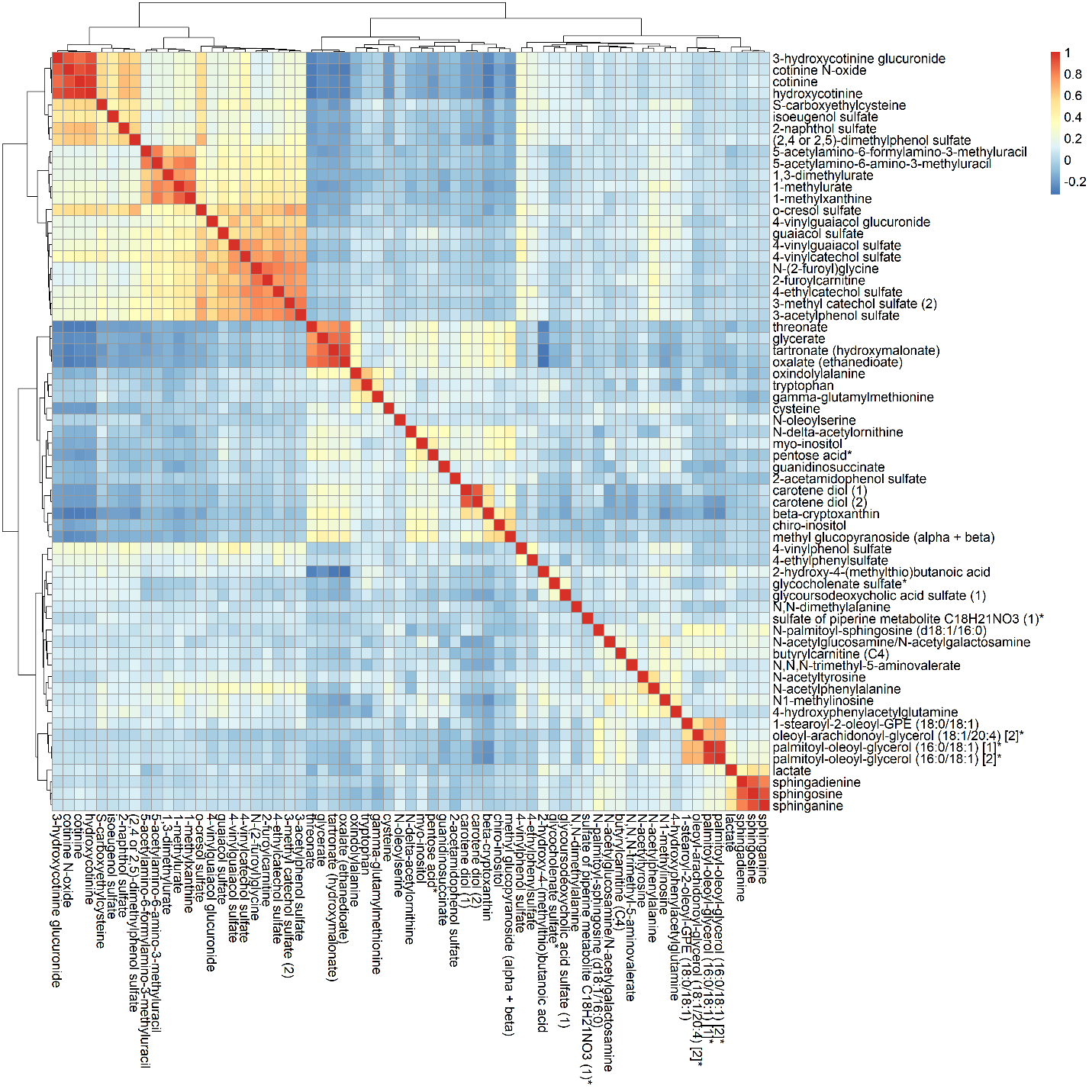
**

**Figure S4**. Agglomerative hierarchical clustering heatmap of the Pearson’s correlation coefficients among the sixty-five metabolites associated with lung cancer risk in ever smokers (FDR $<0.2$).

**
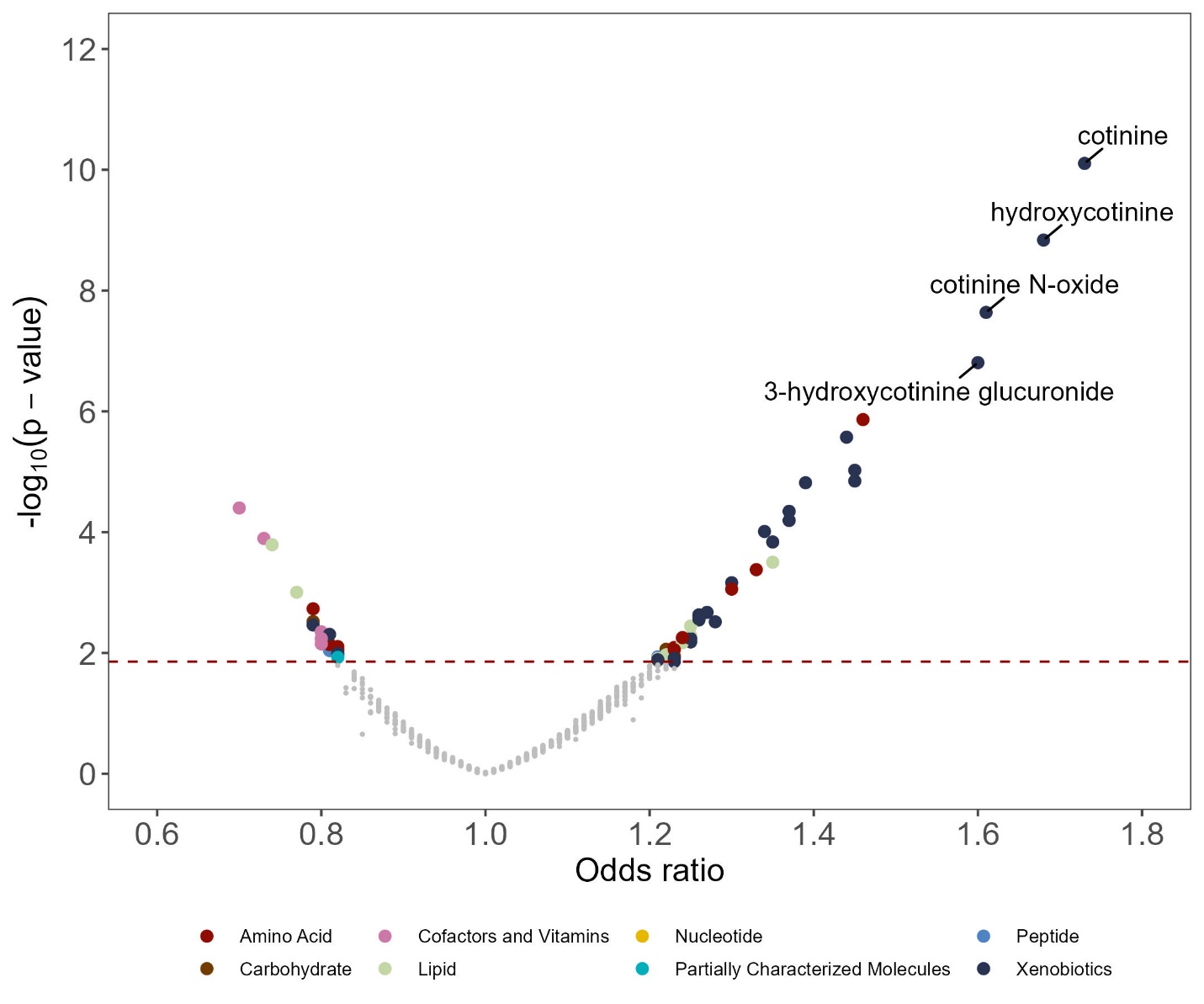
**

**Figure S5.** A volcano plot of associations between metabolites and lung cancer risk in ever smokers. The X-axis denotes the odds ratio of lung cancer-metabolite associations. Odds ratios (95% confidence intervals) per one standard deviation increase in natural log-transformed level of each known metabolite with lung cancer risk were estimated from unconditional logistic regression models, adjusted for age at blood draw, sex, race, date of blood draw, body mass index group, hours since last meal, physical activity, fruits and vegetables consumption, and hormone use. The Y-axis denotes the negative log_10_ of the *p*-value in the lung cancer-metabolite association. Different colors were used to represent different pathways where the metabolites are involved. The dark red dashed line represents FDR < 0.2*.* Four highlighted metabolites are tobacco metabolites.
